# Outcomes for the first four lines of therapy in patients with HER2-positive advanced breast cancer: results from the SONABRE registry

**DOI:** 10.1007/s10549-022-06832-9

**Published:** 2023-01-12

**Authors:** Khava I. E. Ibragimova, Sandra M. E. Geurts, Marissa Meegdes, Frans Erdkamp, Joan B. Heijns, Jolien Tol, Birgit E. P. J. Vriens, Marcus W. Dercksen, Kirsten N. A. Aaldering, Manon J. A. E. Pepels, Linda van de Winkel, Natascha A. J. B. Peters, Nathalie J. A. Teeuwen-Dedroog, Ingeborg J. H. Vriens, Vivianne C. G. Tjan-Heijnen

**Affiliations:** 1 Department of Medical Oncology, GROW, School for Oncology and Developmental Biology, Maastricht University Medical Center, Maastricht, PO BOX 5800, 6202 AZ Maastricht, the Netherlands; 2 Department of Internal Medicine, Zuyderland Medical Center, Sittard-Geleen, the Netherlands; 3 Department of Medical Oncology, Amphia, Breda, the Netherlands; 4 Department of Medical Oncology, Jeroen Bosch Hospital, Den Bosch, the Netherlands; 5 Department of Internal Medicine, Catharina Hospital, Eindhoven, the Netherlands; 6 Department of Medical Oncology, Máxima Medical Center, Eindhoven, the Netherlands; 7 Department of Internal Medicine, Laurentius Hospital, Roermond, the Netherlands; 8 Department of Internal Medicine, Elkerliek Hospital, Helmond, the Netherlands; 9 Department of Internal Medicine, St Anna Hospital, Geldrop, the Netherlands; 10 Department of Internal Medicine, Sint Jans Gasthuis Hospital, Weert, the Netherlands

**Keywords:** Breast neoplasms, Neoplasm metastasis, ERBB2 protein, Registries, Pertuzumab, Treatment Outcome

## Abstract

**Purpose:**

We assessed the systemic treatment choices and outcomes in patients diagnosed with human epidermal growth factor receptor-2-positive (HER2 +) advanced breast cancer (ABC), for the first four lines of systemic therapy and by hormone receptor (HR) status.

**Methods:**

We identified 330 patients diagnosed with HER2 + ABC in 2013–2018 in the Southeast of The Netherlands, of whom 64% with HR + /HER2 + and 36% with HR-/HER2 + disease. Overall survival (OS) from start of therapy was calculated using the Kaplan–Meier method.

**Results:**

In real world, 95% of patients with HR + /HER2 + and 74% of patients with HR-/HER2 + disease received systemic therapy. In HR + /HER2 + disease, use of endocrine, chemo- and HER2-targeted therapy was , respectively, 64%, 46% and 60% in first line, and 39%, 64% and 75% in fourth line. In HR-/HER2 + disease, 91–96% of patients received chemotherapy and 77–91% HER2-targeted therapy, irrespective of line of therapy. In patients with HR + /HER2 + disease, median OS was 34.9 months (95%CI:25.8–44.0) for the first line and 12.8 months (95%CI:10.7–14.9) for the fourth line. In HR-/HER2 + disease, median OS was 39.9 months (95%CI:23.9–55.8) for the first line and 15.2 months (95%CI:10.9–19.5) for the fourth line. For patients treated with first-line pertuzumab, trastuzumab plus chemotherapy, median OS was not reached at 56.0 months in HR + /HER2 + disease and 48.4 months (95%CI:32.6–64.3) in HR-/HER2 + disease.

**Conclusion:**

Survival times for later lines of therapy are surprisingly long and justify the use of multiple lines of systemic therapy in well-selected patients with HER2 + ABC. Our real-world evidence adds valuable observations to the accumulating evidence that within HER2 + ABC, the HR status defines two distinct disease subtypes.

**Supplementary Information:**

The online version contains supplementary material available at 10.1007/s10549-022-06832-9.

## Introduction

The number of treatment options for advanced breast cancer (ABC) has substantially increased over time, especially in patients with human epidermal growth factor receptor-2-positive (HER2 +) disease. Trastuzumab was introduced as the first HER2-targeted therapy, at the end of the nineties of the previous century [1]. During the past two decades, trastuzumab beyond progression (TBP), lapatinib, pertuzumab, T-DM1, neratinib, trastuzumab-deruxtecan, tucatinib and margetuximab were approved by the FDA and/or EMA [2–10]. Until recently, international guidelines recommended that patients with HER2 + ABC are treated with first-line pertuzumab, trastuzumab plus taxane and with second-line T-DM1 regardless of hormone receptor (HR) status [8, 9]. Since 2022, the ASCO guidelines recommend trastuzumab-deruxtecan as preferred second line and tucatinib combined with trastuzumab and capecitabine as third-line treatment option [11]. In the Netherlands, pertuzumab was reimbursed per July 30, 2013, and T-DM1 per June 26, 2014. Access to trastuzumab-deruxtecan and tucatinib is expected soon in the Netherlands, as in many other countries worldwide. In selected patients with HR + /HER2 + disease, endocrine therapy added to trastuzumab or as monotherapy remains an option in patients with low disease burden, long disease-free interval, low performance score, cardiac disease, and personal preference to avoid chemotherapy [12, 13].

Several observational studies have shown that patients diagnosed with HR + /HER2 + disease had a better outcome when compared with patients with HR-/HER2 + disease [14–17]. Recently, we reported that patients with HR-/HER2 + ABC who were diagnosed after the introduction of pertuzumab and T-DM1 had an improved overall survival (OS) as compared with those diagnosed before introduction, whereas survival remained rather similar in patients with HR + /HER2 + ABC [17]. We hypothesized that this might be related to differences in treatment choices in first and subsequent lines of systemic therapy.

To our knowledge, treatment choices per line of therapy and by HR status have not been reported before. Therefore, we studied, in a real-world setting, the delivered systemic therapies per HR status for the first four lines of therapy, and the accompanying patient and tumour characteristics, progression-free survival (PFS) and OS time. These data provide helpful insights for treatment decisions in the future patients, for reimbursement issues and for the design of clinical trials.

## Patients and methods

### Southeast Netherlands advanced breast cancer (SONABRE) registry

Data for this study were obtained from the SONABRE Registry (NCT-03577197). This observational cohort study includes all patients diagnosed with de novo or recurrent ABC in the Southeast of the Netherlands. Information is collected by specially trained registration clerks from medical files including patient and tumour characteristics, treatment information in the curative and palliative setting, response to systemic therapy, and date and cause of death. The Medical Research Ethics Committee of Maastricht University Medical Centre approved the Registry (15-4-239).

### Patients

In this present study, we selected all patients diagnosed with HER2 + ABC in 2013–2018 from nine hospitals, including one academic, five teaching and three non-teaching hospitals. The last follow-up was collected in 2020 and the data lock was on September first, 2020. HER2 positivity was defined as a positive fluorescence in situ hybridization (FISH) result or an immunohistochemistry score of 3 + . HR (oestrogen/progesterone receptor) positivity was defined as positive nuclear staining of ≥ 10% of one or both receptors by immunohistochemistry. To determine the breast cancer subtype, we used information on HR/HER2 status from a metastatic site. If no biopsy of metastatic disease was available, the receptor status was based on the primary tumour or a prior locoregional recurrence.

### Endpoints

We first determined the proportion of patients who received at least one line of palliative systemic therapy and compared their characteristics with patients who received best supportive care only.

Next, we assessed the delivered systemic therapies for the first four lines of therapy with the accompanying patient and tumour characteristics at start of each line of systemic therapy for advanced disease. According to national cancer institute (NCI) criteria [18], a new line of therapy was defined as the introduction of a new systemic agent, with the exception of the introduction of endocrine therapy as maintenance therapy, i.e. in the absence of progression of disease. We estimated the upper and lower limit of the proportion of patients starting a second line to fourth line of therapy, the so-called continuation rate. The lower limit of the continuation rate was defined as the proportion of patients who started a second line to fourth line of therapy during follow-up. Then we determined the proportion of patients who had died before starting a specific line of therapy, thus not being able to continue to a next line of therapy. The upper limit of the continuation rate was then calculated as one minus the proportion of patients who died during a specific line. To account for the time-dependent character of the estimation of the continuation rates, competing risk regression was used considering continuation to a new line of therapy and mortality before continuing to a next of line of therapy (i.e. the attrition rate) as the two ‘competing events’. Of note, in a cohort were all patients are followed up until death, the lower and upper limit of the continuation rate will be equal.

The primary endpoints were the PFS and OS per line of systemic therapy. PFS was calculated as the time from the start of the line of systemic therapy until reported progression of disease or death, whichever occurred first. Patients who stopped treatment (e.g. because of toxicity) but did not switch to a new line of therapy in the absence of progression were followed until progression or death whichever occurred first. Conversely, patients who switched to a next line of therapy in the absence of progression were censored at time of switching. OS was defined as the time from the start of the line of palliative systemic therapy to date of death, or when alive censored at the date of last follow-up.

### Statistical analysis

Baseline patient and disease characteristics for the first four lines of therapy were compared using the Mantel–Haenszel test for trend. PFS and OS per line of therapy were assessed using the Kaplan–Meier methodology per line of therapy, stratified by HR status. Outcomes of patient subgroups with less than 10 patients were not analysed. The *P*-values reported were two-sided and considered statistically significant at a value of ≤ 0.05.

## Results

**Fig. 1:**
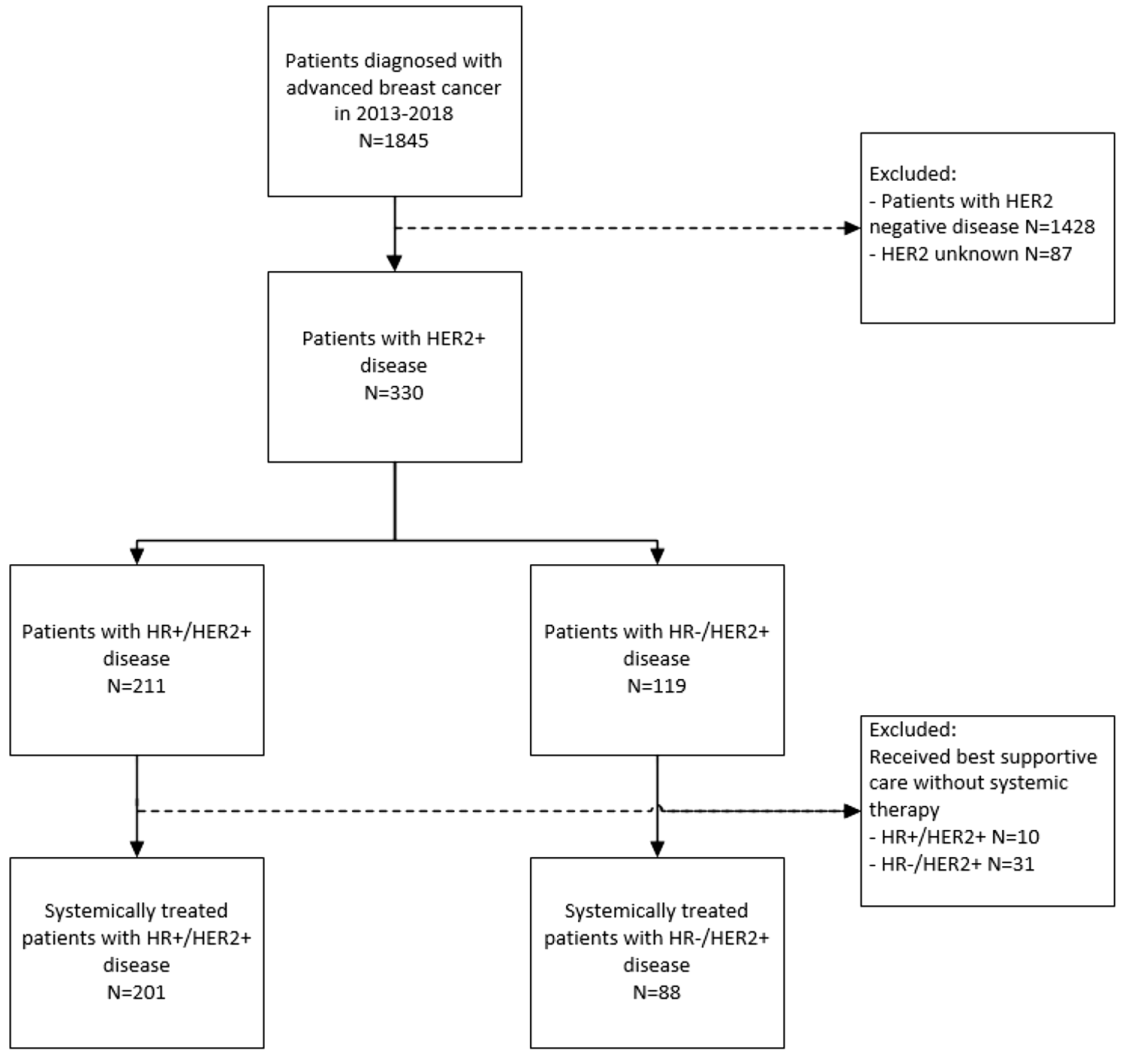
Flowchart patient selection

We identified 330 patients diagnosed with HER2 + ABC in 2013–2018, of whom 211 (64%) with HR + /HER2 + disease and 119 (36%) with HR-/HER2 + disease (Fig. 1). The median follow-up time of systemically treated patients was 47 months (95% confidence interval (CI) 42–52), during which 160 (55%) patients had died, and 7 (2%) patients were lost to follow-up due to transfer to a non-participating hospital.

### Best supportive care only

Ten (5%) patients with HR + /HER2 + and 31 (26%) patients with HR-/HER2 + disease received best supportive care without systemic therapy; they had a median OS of , respectively, 1.0 months (95% CI 0.2–1.9) and 3.0 months (95% CI 2.4–3.7). Patients with HR + /HER2 + disease receiving best supportive care only were older at ABC diagnosis (median 66 vs. 60 years), had a higher rate of WHO performance status 2 + (67% vs 18%) and had a higher rate of central nervous system metastases (30% vs 7%) compared with those who received systemic therapy (Supplementary Table S1). Similarly, patients with HR-/HER2 + disease receiving best supportive care only were older (median 73 vs. 57 years), had more often cardiovascular comorbidity (48% vs 22%), a higher rate of WHO performance status 2 + (68% vs 11%) and a lower rate of soft tissue metastases (26% vs 56%) when compared with those who received systemic therapy (Supplementary Table S1).

### Patient characteristics and outcomes per line of therapy

Among patients who started first-line systemic therapy, an estimated (lower–upper limit) 70–80% of patients with HR + /HER2 + disease started a second line, 51–66% a third line, and 33–54% a fourth line of systemic therapy (Supplementary Figure S1 and Supplementary Table S2). Among HR-/HER2 + patients who started first-line therapy, the estimated continuation rates were 61–78% for second line, 30–61% for third line and 14–50% for fourth line. Patient age, comorbidity (any or cardiovascular), WHO performance status, (neo-)adjuvant systemic therapy use and metastatic-free interval at start of systemic therapy were comparable over the four lines of therapy (Table 1). Though, as expected, the number of metastatic sites increased for each subsequent line of therapy, especially in patients with HR + /HER2 + disease.

**Table 1:** Baseline patient and disease characteristics per line of therapy for systemically treated patients with HER2 + ABC, categorized by HR status

HR + /HER2 +					
Line of therapy	1st line	2nd line	3rd line	4th line	P-value test for trend
Number of patients	*N* = *201*	*N* = *136*	*N* = *88*	*N* = *57*	
	*N (*%)	*N (*%)	*N (*%)	*N (*%)	
Age at start of therapy					
Median age (IQR), years	60 (50–71)	61 (52–71)	60 (51–70)	59 (51–69)	0.88
Comorbidity					
Any	96 (48)	66 (49)	42 (48)	27 (47)	0.99
Cardiovascular	55 (27)	38 (28)	20 (23)	10 (18)	0.38
WHO performance status^a^					0.45
0–1	147 (82)	93 (85)	53 (76)	41 (82)	
≥ 2	32 (18)	16 (15)	17 (24)	9 (18)	
(Neo-)adjuvant therapy^b^					
Any^c^	111 (85)	74 (84)	50 (86)	29 (83)	0.98
HER2-targeted therapy	54 (41)	39 (44)	30 (52)	19 (54)	0.39
Metastatic-free interval					0.81
< 3 months/ de novo	70 (35)	48 (35)	30 (34)	22 (39)	
3–23 months	22 (11)	13 (10)	9 (10)	2 (3)	
≥ 24 months	109 (54)	75 (55)	49 (56)	33 (58)	
Number of metastatic sites					< 0.001
Single	85 (42)	37 (27)	18 (20)	9 (16)	
Multiple	116 (58)	99 (73)	70 (80)	48 (84)	
Metastatic sites^d^					
Bone	138 (69)	103 (76)	71 (81)	51 (90)	0.006
Bone only	48 (24)	22 (16)	11 (13)	7 (12)	0.008
Lymph and Soft tissue^e^	83 (41)	65 (48)	48 (55)	32 (56)	0.09
Visceral^f^	128 (64)	99 (73)	64 (73)	45 (79)	0.08
CNS^g^	13 (7)	14 (10)	16 (18)	9 (16)	0.02

HR-/HER2 +					
Line of therapy	1st line	2nd line	3rd line	4th line	P-value test for trend
Number of patients	*N* = *88*	*N* = *49*	*N* = *22*	*N* = *11*	
	*N (*%)	*N (*%)	*N (*%)	*N (*%)	
Age at start of therapy					
Median age (IQR), years	57 (49–65)	59 (50–66)	54 (47–65)	54 (47–67)	0.95
Comorbidity, any					
Any	34 (39)	19 (39)	8 (36)	6 (55)	0.76
Cardiovascular	19 (22)	9 (18)	3 (14)	2 (18)	0.85
WHO performance status^a^					0.08
0–1	69 (89)	35 (81)	16 (80)	5 (56)	
≥ 2	9 (11)	8 (19)	4 (20)	4 (44)	
(Neo-)adjuvant therapy^b^					
Any^c^	44 (79)	29 (88)	13 (81)	8 (80)	0.75
HER2-targeted therapy	31 (55)	19 (58)	8 (50)	5 (50)	0.95
Metastatic-free interval					0.72
< 3 months/ de novo	32 (36)	16 (33)	6 (27)	1 (9)	
3–23 months	18 (21)	11 (22)	5 (23)	3 (27)	
≥ 24 months	38 (43)	22 (45)	11 (50)	7 (64)	
Number of metastatic sites					
Single	29 (33)	5 (10)	1 (5)	1 (9)	0.001
Multiple	59 (67)	44 (90)	21 (95)	10 (91)	
Metastatic sites^d^					
Bone	46 (52)	30 (61)	14 (64)	7 (64)	0.63
Bone only	8 (9)	2 (4)	1 (5)	1 (9)	0.54
Lymph and Soft tissue^e^	49 (56)	29 (59)	15 (68)	8 (73)	0.57
Visceral^g^	54 (61)	34 (69)	17 (77)	7 (64)	0.50
CNS^g^	14 (16)	19 (39)	7 (32)	4 (36)	0.02

^a^Missing data were excluded; 76 in HR + /HER2 + and 22 in HR-/HER2 +

^b^Among patients with recurrent metastases (excluding patients with de novo ABC)

^c^Any includes chemotherapy, endocrine therapy and targeted therapy

^d^Sum of percentages exceeds 100 because multiple options are possible

^e^Lymph nodes, skin and eye

^f^Liver, lung, pleura, peritoneum, gastrointestinal track, kidney and ovaries

^g^Brain and leptomeningeal

*ABC* advanced breast cancer, *CNS* central nervous system, *HER2* Human Epidermal growth factor Receptor 2, *HR* hormone receptor, *IQR* inter quartile range, *WHO* World Health Organization

**Fig. 2:**
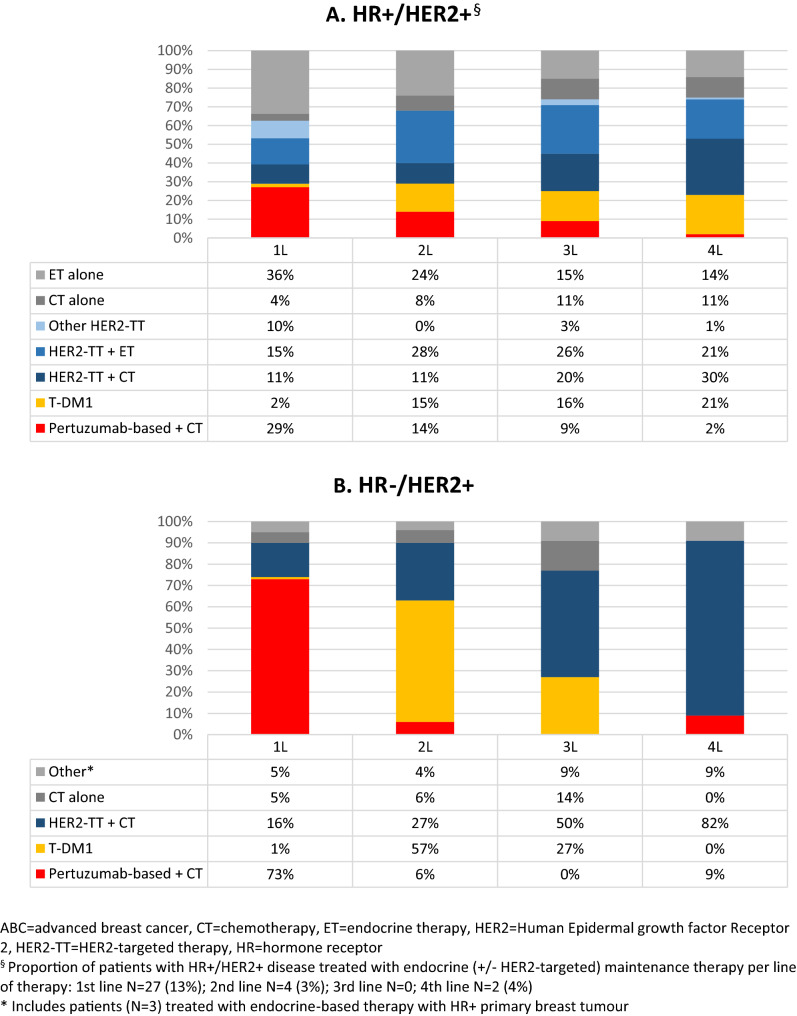
Treatment pattern of patients diagnosed with HER2 + ABC, categorized by HR status. *ABC* advanced breast cancer, *CT* chemotherapy, *ET* endocrine therapy, *HER2* Human Epidermal growth factor Receptor 2, *HER2-TT* HER2-targeted therapy, *HR* hormone receptor. ^§^Proportion of patients with HR + /HER2 + disease treated with endocrine (± HER2-targeted) maintenance therapy per line of therapy: 1st line *N* = 27 (13%); 2nd line *N *= 4 (3%); 3rd line *N* = 0; 4th line *N = *2 (4%). *Includes patients (*N* = 3) treated with endocrine-based therapy with HR + primary breast tumour

In patients with HR + /HER2 + disease, 60% of patients were treated with HER2-targeted therapy in first line, which increased to 75% in fourth line (Fig. 2). In patients with HR-/HER2 + disease, HER2-targeted therapy varied between 77 and 91% in the first four lines of therapy. In general, for the total HER2 + population, once a patient received a HER2-targeted therapy, the next line also contained HER2-targeted therapy in the majority of patients (Supplementary Figure S2). In HR + /HER2 + disease, use of endocrine therapy (with or without HER2-targeted therapy, and including endocrine maintenance therapy after chemotherapy) decreased from 64% in first line to 39% in fourth line, whereas the use of chemotherapy increased from 46% in first line to 64% in fourth line (Fig. 2). In HR-/HER2 + disease, 91–96% of patients received chemotherapy-based therapy, irrespective of line of therapy.

**Fig. 3:**
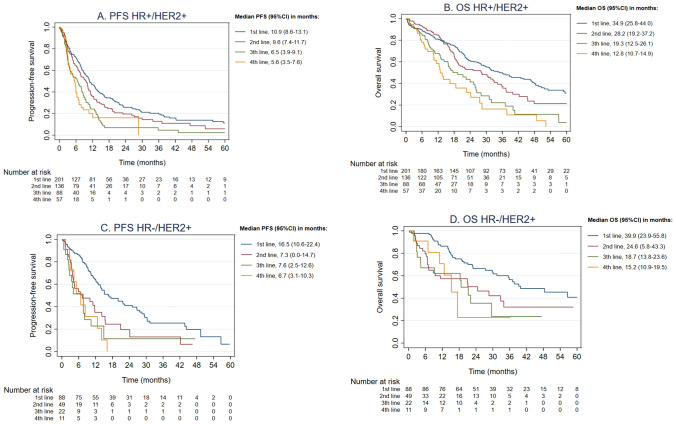
Progression-free survival (PFS) and overall survival (OS) per systemic line of therapy, categorized by HR status

In HR + /HER2 + disease, patients with any first-line systemic therapy (*n* = 201) had a median first-line PFS of 10.9 months (95% CI 8.6–13.1) and a median OS of 34.9 months (95% CI 25.8–44.0), and with any fourth line of therapy (*n* = 57), a median fourth-line PFS of 5.6 months (95% CI 3.5–7.6) and a median OS of 12.8 months (95% CI 10.7–14.9) (Fig. 3A and B).

In HR-/HER2 + disease, patients with any first-line systemic therapy (*n* = 88) had a median first-line PFS of 16.5 months (95% CI 10.6–22.4) and a median OS of 39.9 months (95% CI 23.9–55.8), and with any fourth-line therapy (*n* = 11) had a median fourth-line PFS of 6.7 months (95% CI 3.1–10.3) and a median OS of 15.2 months (95% CI 10.9–19.5) (Fig. 3C and D).

### First-line treatment patterns and outcomes

**Fig. 4:**
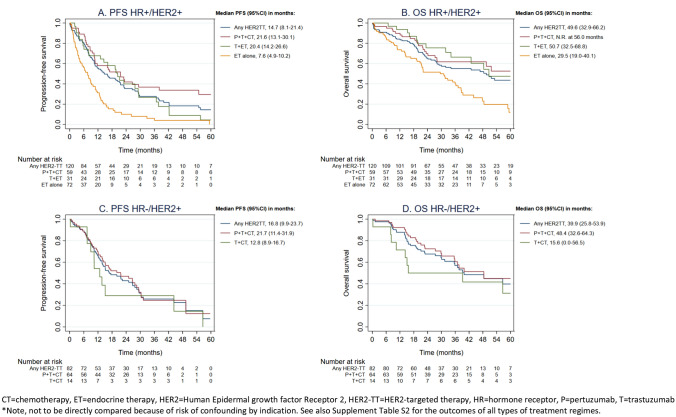
Progression-free survival (PFS) and overall survival (OS) of the most common first-line treatment regimens*, categorized by HR status. *CT* chemotherapy, *ET* endocrine therapy, *HER2* Human Epidermal growth factor Receptor 2, *HER2-TT* HER2-targeted therapy, *HR* hormone receptor, *P* pertuzumab, *T* trastuzumab. *Note, not to be directly compared because of risk of confounding by indication. See also Supplement Table S2 for the outcomes of all types of treatment regimes

In patients systemically treated for HR + /HER2 + disease, the two most common first-line therapies were endocrine monotherapy (*n* = 72, 36%) and pertuzumab, trastuzumab plus chemotherapy (n = 59, 29%) (Fig. 2A, Table 2). Patients treated with first-line endocrine monotherapy had a median first-line PFS of 7.6 months (95% CI 4.9–10.3) and a median OS of 29.5 months (95% CI 19.0–40.1) (Fig. 4). Those treated with first-line pertuzumab, trastuzumab plus chemotherapy had a median first-line PFS of 21.6 months (95% CI 13.1–30.1) and median OS was not reached at 56.0 months. Patients treated with endocrine monotherapy compared with those treated with pertuzumab, trastuzumab plus chemotherapy were older, had more often comorbidity and a worse performance status, a longer metastatic-free interval and more often bone only disease (Table 2).

**Table 2:** Baseline patient and disease characteristics per type of first-line treatment, by HR status

HR + /HER2 +					
First-line treatment	Any HER2-targeted therapy	Pertuzumab-trastuzumab-chemotherapy	Trastuzumab-chemotherapy^h^	Endocrine therapy + trastuzumab	Endocrine monotherapy
Number of patients	120 *N* (%)	59 *N* (%)	21 *N* (%)	31 *N* (%)	72 *N* (%)
Age					
Median age (IQR)	54 (47–64)	51 (44–61)	56 (50–71)	60 (53–78)	68 (60–80)
≥ 70 years	21 (17)	3 (5)	5 (24)	13 (42)	28 (39)
Comorbidity					
Any	45 (37)	19 (32)	5 (24)	16 (52)	47 (65)
Cardiovascular	24 (20)	8 (14)	4 (19)	11 (36)	29 (40)
WHO performance status^a^					
0–1	93 (87)	49 (89)	11 (69)	26 (93)	47 (72)
≥ 2	14 (13)	6 (11)	5 (31)	2 (7)	18 (28)
(Neo-)adjuvant therapy^b^					
Any^c^	59 (86)	24 (83)	9 (82)	20 (87)	48 (83)
HER2-targeted therapy	37 (54)	16 (55)	4 (36)	11 (48)	16 (28)
Metastatic-free interval					
< 3 months/ de novo	51 (43)	30 (51)	10 (48)	8 (26)	14 (20)
3–23 months	13 (11)	2 (3)	1 (4)	8 (26)	6 (8)
≥ 24 months	56 (47)	27 (46)	10 (48)	15 (48)	52 (72)
Number of metastatic sites					
Single	49 (41)	22 (37)	8 (38)	16 (52)	31 (43)
Multiple	71 (59)	37 (63)	13 (62)	15 (48)	41 (57)
Metastatic sites^d^					
Bone	77 (64)	38 (64)	11 (52)	23 (74)	55 (76)
Bone only	22 (18)	10 (17)	0 (0)	11 (36)	22 (31)
Lymph and Soft tissue^e^	51 (43)	27 (46)	10 (48)	9 (29)	28 (39)
Visceral^f^	83 (69)	44 (75)	18 (86)	16 (52)	40 (56)
CNS^g^	8 (7)	3 (5)	3 (14)	0 (0)	5 (7)

HR-/HER2 +			
First-line treatment	Any HER2-targeted therapy	Pertuzumab-trastuzumab-chemotherapy	Trastuzumab-chemotherapy^h^
Number of patients	82 *N* (%)	64 *N* (%)	14 *N* (%)
Age			
Median age (IQR)	56 (48–63)	57 (49–62)	59 (49–78)
≥ 70 years	15 (18)	10 (16)	5 (36)
Comorbidity, any			
Any	30 (37)	25 (39)	5 (36)
Cardiovascular	17 (21)	13 (20)	4 (29)
WHO performance status^a^			
0–1	64 (88)	53 (90)	8 (73)
≥ 2	9 (12)	6 (10)	3 (27)
(Neo-)adjuvant therapy^b^			
Any^c^	41 (80)	29 (85)	8 (62)
HER2-targeted therapy	30 (59)	20 (59)	6 (46)
Metastatic-free interval			
< 3 months/ de novo	31 (38)	30 (47)	1 (7)
3–23 months	15 (18)	7 (11)	5 (36)
≥ 24 months	36 (44)	27 (42)	8 (57)
Number of metastatic sites			
Single	27 (33)	20 (31)	5 (36)
Multiple	55 (67)	44 (69)	9 (64)
Metastatic sites^d^			
Bone	44 (54)	33 (52)	9 (64)
Bone only	8 (10)	6 (9)	2 (6)
Lymph and Soft tissue^e^	46 (56)	36 (56)	8 (57)
Visceral^f^	49 (60)	42 (66)	6 (43)
CNS^g^	14 (17)	10 (16)	2 (14)

^a^Missing data were excluded

^b^Among patients with recurrent metastases (excluding patients with de novo ABC)

^c^Any includes chemotherapy, endocrine therapy and targeted therapy

^d^Sum of percentages exceeds 100 because multiple options are possible

^e^Lymph nodes, skin and eye

^f^Liver, lung, pleura, peritoneum, gastrointestinal track, kidney and ovaries

^g^Brain and leptomeningeal

^h^Among the 35 patients treated with trastuzumab plus chemotherapy, 26 (75%) were diagnosed in 2013–2014 and 9 (25%) in 2015–2018

*ABC* advanced breast cancer, *CNS* central nervous system, *HER2* Human Epidermal growth factor Receptor 2, *HR* hormone receptor, *IQR* inter quartile range, *WHO* World Health Organization

Among patients systemically treated for HR-/HER2 + disease, the two most common first-line therapies were pertuzumab, trastuzumab plus chemotherapy (*n* = 64, 73%) and trastuzumab plus chemotherapy without pertuzumab (n = 14, 16%) (Fig. 2B, Table 2). Patients treated with first-line pertuzumab, trastuzumab plus chemotherapy had a median PFS of 21.7 months (95% CI 11.4–31.9) and a median OS of 48.4 months (95% CI 32.6–64.3) (Fig. 4). Patients treated with first-line trastuzumab plus chemotherapy had a median PFS of 12.8 (95% CI 8.9–16.7) and a median OS of 15.6 (95% CI 0.0–56.5) months, respectively (Fig. 4). Patients treated with trastuzumab plus chemotherapy were older, had a worse performance status and less often de novo metastases when compared with patients treated with pertuzumab, trastuzumab plus chemotherapy (Table 2).

## Discussion

Real-world data support daily clinical decision-making and future study designs, in addition to data from randomized controlled trials. It is therefore important to gain insight into the characteristics of patients we treat in real-life, the (number and type of) lines of therapy they receive, and the outcomes of delivered treatment per line of therapy and per HR status. We collected the data of patients diagnosed with HER2 + ABC in the Southeast of The Netherlands in the years 2013–2018, a time frame in which pertuzumab and T-DM1 became available. We showed that in real world, 5% of patients with HR + /HER2 + disease and 26% of patients with HR-/HER2 + disease received best supportive care without systemic therapy. These patients had poor baseline characteristics and a very poor outcome. With the exception of an increase in the number of metastatic sites for later lines of therapy, patient characteristics at start of therapy were largely independent of line of therapy. As a result of patient selection, the proportion of patients continuing to a next line of therapy gradually declined, whereby the majority continued on HER2-targeted therapy once started. This was the case irrespective of the HR status. Chemotherapy use was significantly lower in patients treated for HR + /HER2 + versus HR-/HER2 + ABC, in all lines of therapy. Use of endocrine-based therapy was common in patients with HR + /HER2 + disease. Median survival times for later lines of therapy were surprisingly long, justifying the use of multiple lines of systemic therapy in well-selected patients with HER2 + ABC.

We observed in our study that only a small proportion of patients with HR + /HER2 + ABC received best supportive care only as compared with a much larger proportion of patients with HR-/HER2 + disease. This may indicate that some patients with HR + /HER2 + disease were fit enough for endocrine therapy but not for chemotherapy plus HER2-targeted therapy. In other patients, endocrine therapy without trastuzumab may have been chosen because trastuzumab use would have obstructed reimbursement of pertuzumab use in the next line of therapy [19, 20]. For patients with HR + /HER2 + ABC who are fit enough for chemotherapy, first-line pertuzumab, trastuzumab plus chemotherapy, followed by maintenance endocrine and trastuzumab therapy is nowadays considered the best treatment option [11].

Only few other investigators studied the use of multiple lines of systemic therapy in patients with HER2 + ABC [21–23]. Consistent with their observations, we also found that the majority of patients received HER2-targeted therapy in first and subsequent lines of therapy, illustrating the high use of trastuzumab beyond progression. In addition, we showed that in daily practice, 36% of patients with HR + /HER2 + disease received initial endocrine therapy as monotherapy and 15% received endocrine therapy combined with HER2-targeted therapy. This high use of endocrine monotherapy was also observed in two multicentre studies in the United States of America (SystHERs and Flatiron: 59% and 24% in first line) [21–23]. A lower number was reported for a European retrospective cross-sectional study, where only 1% of registered patients were treated with endocrine monotherapy, potentially due to patient selection [21]. Noteworthy, we observed that a large proportion of patients with HR + /HER2 + disease received also endocrine therapy during later lines of therapy.

Considering patients treated with any systemic therapy, we observed a median OS of 34.9 months in HR + /HER2 + and a median OS of 39.9 months in HR-/HER2 + disease. When only looking at patients who were treated with first-line pertuzumab, trastuzumab plus chemotherapy, we noted a comparable median PFS (i.e. respectively, 21.6 and 21.7 months), whereas median OS differed (i.e. respectively, not reached at 56 months and 48.4 months). The PFS data confirm that pertuzumab is comparably effective in HR + /HER2 + and HR-/HER2 + disease. The better OS outcome in HR + /HER2 + disease may be the result of different biology and/or by the additional availability of endocrine (maintenance) therapy. It also stresses the impact of patient selection in daily clinical practice. If we select only the fittest patients for the more toxic and more expensive treatments, we offer more cost-effective care. In a time frame where health care costs are continually at the rise and the number of available health care professionals is in decline, we need to consider careful patient selection. Of note, the outcomes of patients selected for pertuzumab in our cohort are in line with the results found in the registration CLEOPATRA trial [10]. We therefore conclude that our patient selection for pertuzumab-based therapy was appropriate.

Preclinical and clinical studies have suggested that the use of dual blockade of the oestrogen receptor (ER) and HER2 receptor for patients with HR + /HER2 + disease prevents relative therapy resistance [24–28]. The use of HER2-targeted therapy in HR + /HER2 + disease is associated with an up-regulation of ER expression and subsequently decreasing HER2 signal activity. Vice versa, the use of endocrine therapy is associated with an increased HER2 signal activity potentially leading to a downregulation of both ER and progesterone receptor (PR) expression. As a result, blocking only one receptor leads to resistance and reduced treatment efficacy. One could argue that patients treated with endocrine therapy alone in second line and beyond missed out on important treatment benefits of adding HER2 blockage. Recently, the MonarcHER phase II trial reported that CDK4/6 inhibitor plus endocrine therapy in combination with trastuzumab was superior to chemotherapy plus trastuzumab in patients who had previously received at least two lines of HER2-targeted therapies for ABC, whereas CDK4/6 inhibitor plus trastuzumab without endocrine therapy was comparable to chemotherapy plus trastuzumab [29]. More studies need to investigate the role of combining dual endocrine targeted and dual HER2-targeted therapy in patients with HR + /HER2 + disease.

The strength of our prospective cohort study lies in the inclusion of all patients diagnosed with HER2 + ABC from a recent five-year inclusion period showing a more current treatment pattern over multiple lines of therapy with a significant follow-up time. We were also able to identify patients not systemically treated. These patients are generally not included in observational studies. The data were manually screened and collected by specially trained registration clerks, which contributed to the high quality of the data. Inherent to the observational character of the study, effectiveness comparison between lines and type of therapy was not possible due to the risk of confounding by indication affecting treatment decisions and prognosis. The reasoning behind chosen treatment options was generally not documented in the medical files. In addition, the evaluation of progressive disease was based on the physician’s assessment which did not always include imaging. Lastly, the number of patients with HR-/HER2 + disease receiving a fourth-line therapy (n = 11) is limited, making it difficult to reliably interpret the results. Yet, it is an important addition to the evolving evidence that HR + /HER2 + and HR-/HER2 + ABC are to be considered as two distinct diseases. These study findings provide important mirror information for the treating physicians, and it is required as model input for the design of new clinical trials and the reimbursement, implementation and evaluation of new systemic drugs.

In conclusion, we studied the real-life treatment choices and outcomes of patients diagnosed in 2013–2018 with HER2-positive ABC. We showed that patients with maintenance of a good performance status received multiple lines of (HER2-targeted) therapies with even in the fourth line, a median overall survival of more than one year, which is a reassuring observation. Endocrine monotherapy use was in line with guideline recommendations and associated with older age and poorer performance status. In patients with HR + /HER2 + disease, endocrine-based therapy was often given over the subsequent lines of therapy. In the future, chemotherapy-free regimens consisting of multiple targeted therapies are expected. The distinction by HR status within the HER2 + ABC subtype will become increasingly important.

## Supplementary Information

Below is the link to the electronic supplementary material.

Supplementary file1 (DOCX 467 kb)

## Data Availability

Data are available upon request (vcg.tjan.heijnen@mumc.nl).
