# Supplementary material for: Outcomes for the first four lines of therapy in patients with HER2-positive advanced breast cancer: results from the SONABRE registry
Source: Breast Cancer Res Treat. 2023 Jan 12;198(2):239–51. doi: 10.1007/s10549-022-06832-9 (PMC10020272; doi:10.1007/s10549-022-06832-9)

**Supplementary Table S1.** Baseline characteristics of patients with HER2-positive advanced breast cancer receiving best supportive care without systemic treatment compared with patients receiving at least one line of palliative systemic therapy, categorized by HR-status

|  | **HR+/HER2+** | | | **HR-/HER2+** | | |
| --- | --- | --- | --- | --- | --- | --- |
|  | **Best supportive**  **care only** | **Systemically**  **treated** |  | **Best supportive**  **care only** | **Systemically**  **treated** |  |
| Number of patients | **N=10** | **N=201** |  | **N=31** | **N= 88** |  |
| **Characteristics** | *N (%)* | *N (%)* | *P-value* | *N (%)* | *N (%)* | *P-value* |
| **Age at diagnosis ABC** |  |  |  |  |  |  |
| Median (IQR) , years | 66 (57-76) | 60 (50-70) | 0.04 | 73 (61-85) | 57 (49-65) | <0.001 |
| ≥70 years | 4 (40) | 51 (25) | 0.30 | 18 (58) | 16 (18) | <0.001 |
| **Comorbidity** |  |  |  |  |  |  |
| Any | 5 (50) | 96 (48) | 0.89 | 20 (65) | 34 (39) | 0.13 |
| Cardiovascular | 3 (30) | 55 (27) | 0.86 | 15 (48) | 19 (22) | 0.005 |
| **WHO performance score**^×^ |  |  | <0.001 |  |  | <0.001 |
| WHO 0-1 | 3 (33) | 147 (82) |  | 8 (32) | 69 (89) |  |
| WHO ≥2 | 6 (67) | 32 (18) |  | 17 (68) | 9 (11) |  |
| **(Neo-)adjuvant therapy**^§^ |  |  |  |  |  |  |
| Any^¥^ | 5 (83) | 111 (85) | 0.93 | 16 (64) | 44 (79) | 0.17 |
| HER2-targeted therapy | 3 (50) | 54 (41) | 0.67 | 13 (52) | 31 (55) | 0.78 |
| **Metastatic-free interval** |  |  | 0.52 |  |  | 0.22 |
| <3 months/ *de novo* | 4 (40) | 70 (35) |  | 6 (19) | 32 (36) |  |
| 3-23 months | 0 (0) | 22 (11) |  | 8 (26) | 18 (21) |  |
| ≥ 24 months | 6 (60) | 109 (54) |  | 17 (55) | 38 (43) |  |
| **Number initial metastatic**  **sites** |  |  | 0.16 |  |  | 0.13 |
| Single organ | 2 (20) | 85 (42) |  | 15 (48) | 29 (33) |  |
| Multiple organ | 8 (80) | 116 (58) |  | 16 (52) | 59 (67) |  |
| **Metastatic sites**^‡^ |  |  |  |  |  |  |
| Bone | 6 (60) | 138 (69) | 0.57 | 15 (48) | 46 (52) | 0.71 |
| Bone only | 1 (10) | 48 (24) | 0.31 | 3 (10) | 8 (9) | 0.92 |
| Lymph and Soft tissue* | 5 (50) | 83 (41) | 0.59 | 8 (26) | 49 (56) | 0.004 |
| Visceral** | 7 (70) | 128 (64) | 0.69 | 15 (48) | 54 (61) | 0.21 |
| CNS*** | 3 (30) | 13 (7) | 0.006 | 9 (29) | 14 (16) | 0.11 |

ABC= advanced breast cancer, CNS=central nervous system, HR=hormone receptor, HER2=Human Epidermal growth factor Receptor 2, IQR= interquartile range, WHO=World Health Organization

^×^ Missing data were excluded; 23 in HR+/HER2+ and 16 in HR-/HER2+; ^§^ Among patients with recurrent metastases (excluding patients with *de novo* ABC); ^¥^Any includes chemotherapy, endocrine therapy and targeted therapy; ^‡^ Sum of percentages exceeds 100 because multiple options are possible; ^*^ Lymph nodes, skin and eye; ^**^ Liver, lung, pleura, peritoneum, gastrointestinal track, kidney and ovaries; ^***^ Brain and leptomeningeal

**Supplementary Figure S1.** Continuation and competing mortality rates per line of therapy among patients receiving systemic therapy for HR+/HER2+ and HR-/HER2+ ABC

| 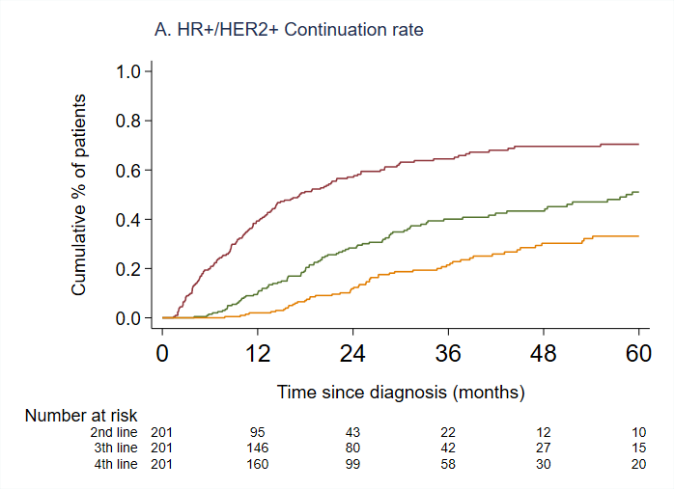 | 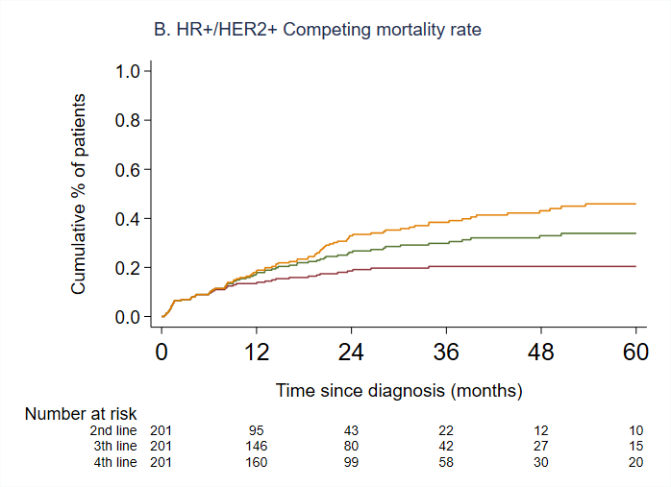 |
| --- | --- |
| 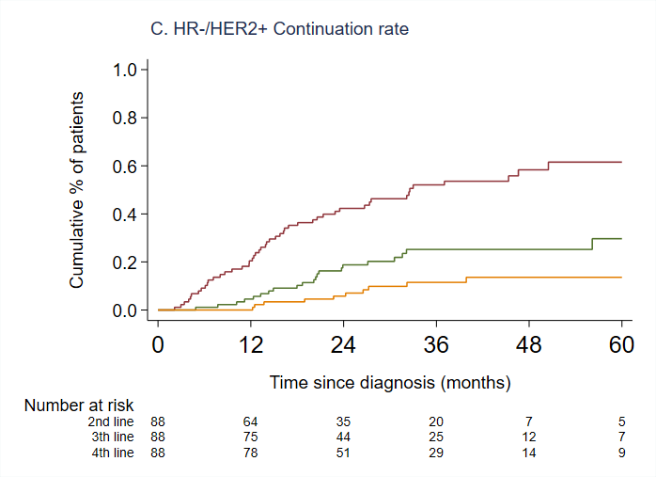 | 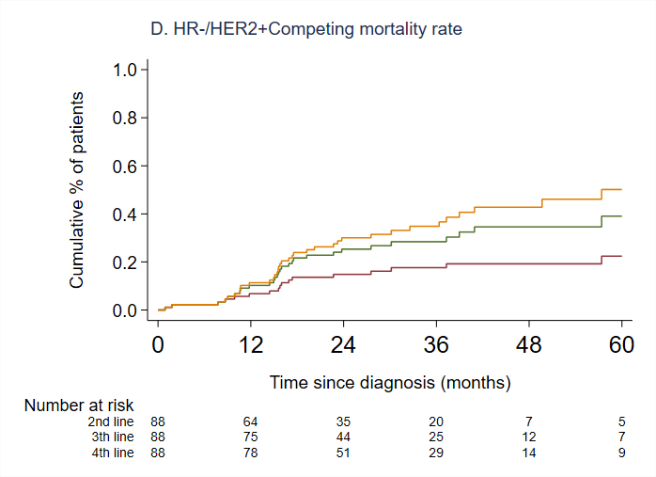 |

The number at risk includes the number of patients who did not start the line of interest nor died and were still in follow-up.

See Supplementary Table S2 for the rates at 60 months of follow-up.

Red=second treatment line

Green= third treatment line

Yellow= Fourth treatment line

**Supplementary Table S2.** Lower and upper limit of the continuation rates of patients starting a second- to fourth-line of therapy *at five-years of follow-up* in HR+/HER2+ and HR-/HER2+ ABC

| **Line of therapy** | **Observed continuation rate**  **(i.e. lower limit)** | **Observed competing mortality rate**  **(i.e. 1-upper limit)** | **Still elligible** | **Estimated continuation rate**  **Lower – upper limit*** |
| --- | --- | --- | --- | --- |
| **HR+/HER2+** |  |  |  |  |
| 2^nd^ line | 70% | 20% | 10% | 70%-80% |
| 3^th^ line | 51% (73% of 2^nd^ line) | 34% | 15% | 51%-66% (73%-83% of 2^nd^ line) |
| 4^th^ line | 33% (65% of 3^th^ line) | 46% | 21% | 33%-54% (65%-82% of 3^th^ line) |
| **HR-/HER2+** |  |  |  |  |
| 2^nd^ line | 61% | 22% | 17% | 61%-78% |
| 3^th^ line | 30% (49% of 2^nd^ line) | 39% | 31% | 30%-61% (49%-78% of 2^nd^ line) |
| 4^th^ line | 14% (47% of 3^th^ line) | 50% | 36% | 14%-50% (47%-82% of 3^th^ line) |

* In a cohort were all patients are followed up until death, the sum of the continuation rate and compering mortality rate will reach 100%, then the lower and upper limit of the continuation rate will be equal.

**Supplementary Table S3.** The outcome of first-line therapies in patients with HER2+ ABC, categorized by HR-status

|  | **Type of therapy** | **Number of**  **patients** | **PFS^§^ in months**  Median (95% CI) | **OS^§^ in months**  Median (95% CI) |
| --- | --- | --- | --- | --- |
| **HR+/HER2+** | Any HER2-targeted therapy | 120 | 14.7 (8.1-21.4) | 49.6 (32.9-66.2) |
|  | Pertuzumab + trastuzumab + chemotherapy | 59 | 21.6 (13.1-30.1) | N.R. at 56.0 months |
|  | Trastuzumab + chemotherapy | 21 | 9.3 (6.2-12.5) | 22.5 (11.9-33.1) |
|  | Trastuzumab + endocrine therapy | 31 | 20.4 (14.2-26.6) | 50.7 (32.5-68.8) |
|  | Chemotherapy alone | 9 | N.A. | N.A. |
|  | Endocrine monotherapy | 72 | 7.6 (4.9-10.2) | 29.5 (19.0-40.1) |
| **HR-/HER2+** | Any HER2-targeted therapy | 82 | 16.8 (9.9-23.7) | 39.9 (25.8-53.9) |
|  | Pertuzumab + trastuzumab + chemotherapy | 64 | 21.7 (11.4-31.9) | 48.4 (32.6-64.3) |
|  | Trastuzumab + chemotherapy | 14 | 12.8 (8.9-16.7) | 15.6 (0.0-56.5) |
|  | Trastuzumab + endocrine therapy | 0 | N.A. | N.A. |
|  | Chemotherapy alone | 4 | N.A. | N.A. |
|  | Endocrine monotherapy | 2 | N.A. | N.A. |

ABC=advanced breast cancer, HER2=Human Epidermal growth factor Receptor 2, HR=hormone receptor, N.A.=not analysed because of low number of patients (<10), N.R.=not reached, OS=overall survival, PFS=progression-free survival

§ From start of systemic therapy

**Supplementary Figure S2.** Treatment sequencing from the first- to fourth-line of therapy for all patients with HER2+ ABC (irrespective of HR-status because of too many treatment options)


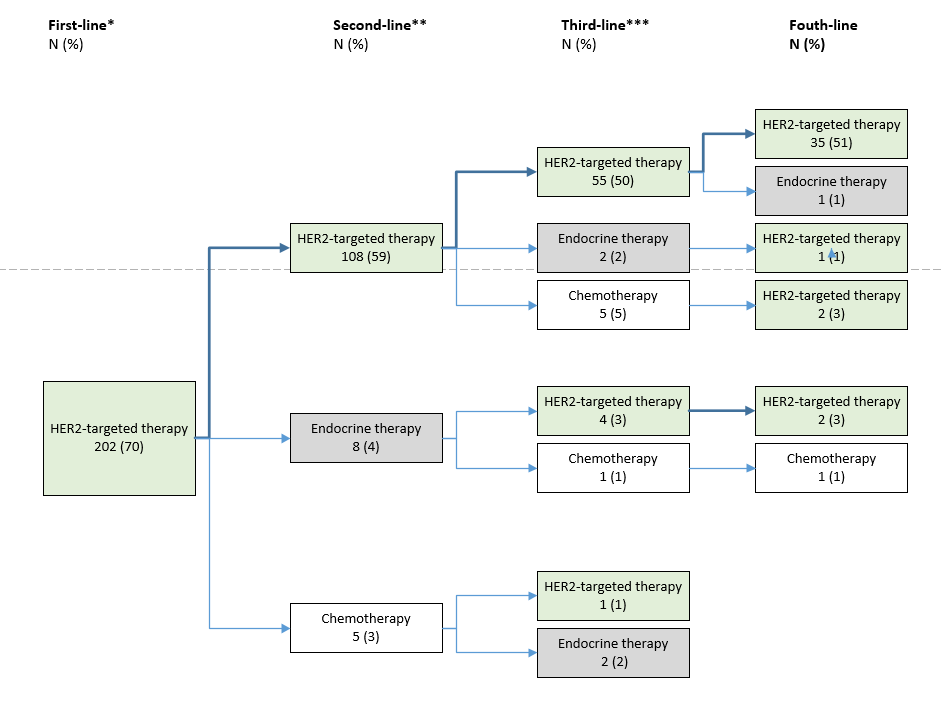


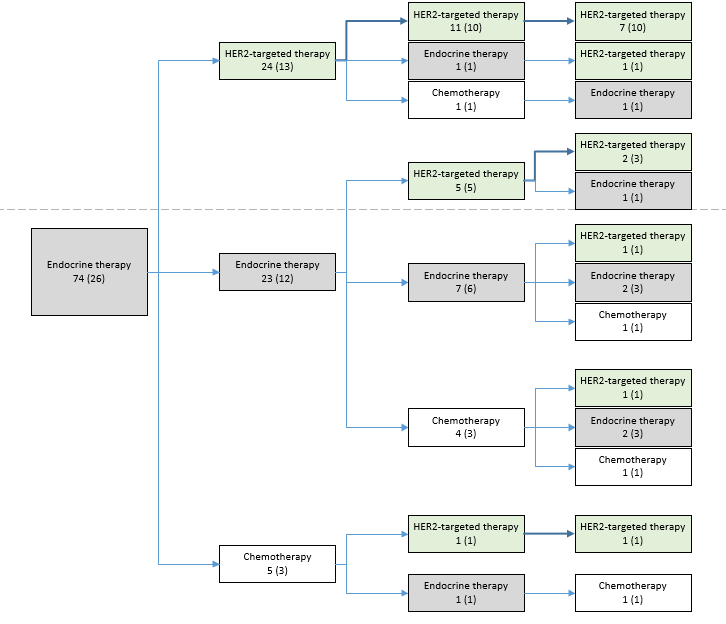


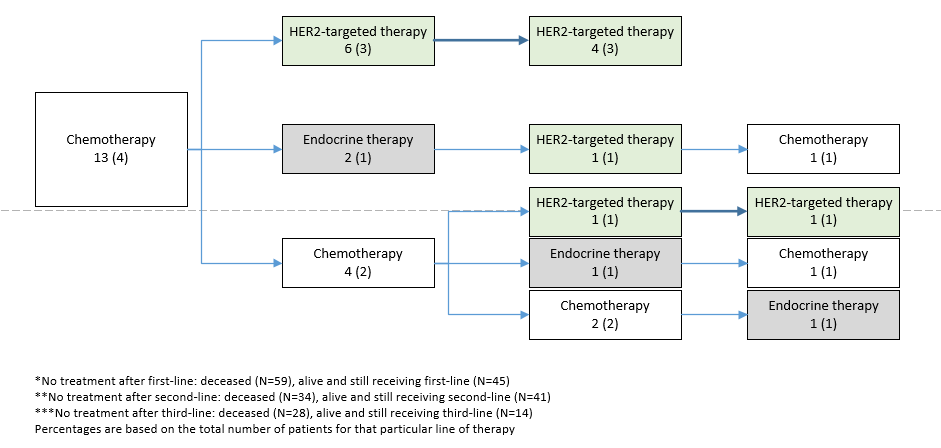

Supplement: Supplementary file 1 — Supplementary file1 (DOCX 467 kb) [file 10549_2022_6832_MOESM1_ESM.docx]
